# Bioaccumulation of Mineral Elements in Different Biological Substrates of Athletic Horse from Messina, Italy

**DOI:** 10.3390/ani10101877

**Published:** 2020-10-14

**Authors:** Francesco Fazio, Enrico Gugliandolo, Vincenzo Nava, Giuseppe Piccione, Claudia Giannetto, Patrizia Licata

**Affiliations:** 1 Department of Veterinary Science, University of Messina, Polo SS Annunziata, 98168 Messina, Italy; gpiccione@unime.it (G.P.); clgiannetto@unime.it (C.G.); plicata@unime.it (P.L.); 2 Department of Chemical, Biological, Pharmaceutical and Environmental Science, University of Messina, 98166 Messina, Italy; egugliandolo@unime.it; 3 BioMorf Department, University of Messina, Polo SS Annunziata, 98168 Messina, Italy; vnava@unime.it

**Keywords:** athletic horse, bioaccumulation, Milazzo, mineral elements, toxicological risk

## Abstract

**Simple Summary:**

The objective of this study was to evaluate the levels and potential bioaccumulation of vanadium (V), chromium (Cr), cobalt (Co), copper (Cu), zinc (Zn), cadmium (Cd), lead (Pb), and bismuth (Bi) in horses from the industrial risk area of Sicily (Italy). Different biological substrates (whole blood, serum, tail, and mane) and samples of hay, concentrate, and water were analyzed. Pearson’s test was applied to evaluate the correlation of mineral concentrations between whole blood and serum, and between tail and mane. The results showed statistical differences in tested mineral elements among the biological substrates. Minerals had a non-homogenous distribution in the organism, showing different concentrations in the biological substrates.

**Abstract:**

The objective of this study was to evaluate the levels and the potential bioaccumulation of vanadium (V), chromium (Cr), cobalt (Co), copper (Cu), zinc (Zn), cadmium (Cd), lead (Pb), and bismuth (Bi) in horses from the industrial risk area of Sicily (Italy). Different biological substrates (whole blood, serum; tail and mane) and samples of hay, concentrate and water provided to the horses were processed by means of Thermo Scientific iCAP-Q ICP–MS spectrometer for mineral concentration. One-way analysis of variance (ANOVA) was applied to show the differences in various trace elements in the biological substrates. Pearson’s test was applied to evaluate the correlation of mineral concentrations between whole blood and serum; and tail and mane. The results showed statistical differences of tested mineral elements among biological substrates; Cr whole blood concentrations were negatively correlated with serum concentrations and a positive correlation between whole blood and serum was observed for Cd and Bi. This latter also showed a positive correlation between mane and tail. The concentrations of V, Cr, and Pb in tail with serum and whole blood samples were negatively correlated, while the concentrations of Cd in tail and serum samples were positively correlated. Minerals had a non-homogenous distribution in the organism, showing different concentrations in the biological substrates.

## 1. Introduction

Minerals are essential trace elements for the survival of living beings, which are divided on the basis of their body concentrations as macro- and microminerals. When their physiological values are exceeded, they become toxic for the organisms themselves. The diffusion and stability of mineral elements in the environment make them unique as pollutants or essential dietary components. They are neither created nor destroyed by chemical processes but are redistributed in the environment, and the amount of mineral elements in water and plants is influenced by the composition of the atmosphere. In recent years, atmospheric composition has been highly influenced by urbanization, industrialization, and the use of chemical fertilizers and soil correctives [1,2]. Compounds and metal alloys, along with other elements, are essential materials in the contemporary world [3]. Trace minerals play a vital role in the health and development of horses and some are contained in grass, while others can be added as supplements to hay or cereal products to provide one or more specific nutrients. To date, many studies have been conducted to evaluate the acute effects of mineral element and trace element poisoning, and non-acute exposure over long periods of time can induce several effects [4,5]. Different soft tissue, urine, hair, whole blood, and serum can be used to evaluate the concentration of minerals in animal organisms, and can also be used as indicators of environmental degradation status [6]. In the body, mineral elements have a different affinity for each substrate on the basis of the chemical compounds in the substrate and, in particular, a different distribution between cellular and noncellular compartments has been observed in blood [7]. Hair analysis has been used to monitor human and animal exposure to mineral elements, as reported by Ward and Savage [8] and Patra et al. [9]. By contrast, some authors have reported ineffective use of serum, blood, and hair as indicators of environmental pollution [10]. Milazzo is an area of Sicily that has a high degree of pollution due to the presence of large refinery plants, a power plant, and other factories. Recent studies have reported mercury bioaccumulation in blood and serum samples of horses in this area [11]. On the basis of this, we investigated the distribution of V, Cr, Co, Cu, Zn, Cd, Pb, and Bi in several biological components (whole blood, serum; tail and mane) in horses located at an equine center near Milazzo (Messina, Italy). Moreover, in all horses, we studied the possible correlations of the minerals concentrations among the various biological substrates (serum, whole blood, tail, and mane).

## 2. Materials and Methods

### 2.1. Animals

This work complied with EU Directive 2010/63/EU regarding the protection of animals used for scientific purposes, and with current Italian legislation (Directive 2010/63/EU). Twenty clinically healthy Italian Saddle geldings aged 8–11 years (9.3 ± 1.7 years) and with an average body weight of 460 ± 20 kg were enrolled in this study during winter 2019 (air temperature 12 °C and relative humidity 66%). All horses were subjected to a regular training program which included six days of work during the week. Each training session was about 1 h in duration and included walking, trotting, and galloping. Two days a week, some little jumps were added, and one day a week, an obstacle course containing 10 jumps with a height of 1.30 height was completed. With respect to health status, clinical examination (evaluation of body temperature, heart rate, respiratory rate), routine hematology, and biochemistry were evaluated (data not shown). All horses lived at the same horse training center, which is located near the industrialized area of Milazzo (Messina, Sicily 38°00′49″ N 15°25′18″ E, 80 m above sea level). The horses were conventionally fed three times a day (06:30, 12:00, and 19:30) with good-quality hay (2.0 kg/100 kg body weight) and concentrate (1.0 kg/100 kg body weight); water was available ad libitum. The horses were fed standard rations consisting of hay (first-cut meadow hay, sun-cured, late-cut, and a mixture of cereals), and 50% each of oats and barley. The analytical parameters of the concentrate were as follows: 13.2% crude protein, 3.1% crude oils and fats, 11.5% crude cellulose, 8.0% crude ash, 0.2% sodium, 1.2% calcium, and 0.6% phosphorous. The evaluation of the minerals present in the hay and concentrate is reported in Table 1. 

#### 2.1.1. Sample Collection

For all horses, blood samples were collected before the morning ration and far from physical exercise at 06:00 through jugular venipuncture, using vacutainer tubes (Terumo Corporation, Tokyo, Japan) with K3-EDTA (EDTA, ethylenediamine tetraacetic acid) and without additives. Samples in K3-EDTA were stored at refrigerated temperature (4 °C); and samples without anticoagulant additive were centrifuged (2500× *g* for 10 min) and sera were stored at −20 °C until analysis. At the same time, the mane and tail were excised with rigid plastic scissors and stored in plastic bags for analysis. Mane was collected in the middle of the neck close to the skin, and tail from where it hung. Hay, concentrate, and water were collected in plastic tanks and stored at 4 °C.

#### 2.1.2. Samples Analysis 

Each sample was accurately weighed into acid-prewashed PTFE (polytetrafluoroethylene) vessels and analyzed by mass spectrometry with inductively ICP-MS iCAP-Q (Thermo Fisher Scientific, Waltham, MA, USA) for determination of V, Cr, Co, Cd, Pb, and Bi and by emission spectrometry with inductively ICP-OES ULTIMA 2 ICP-OES (HORIBA, Kyoto, Japan) for Zn and Cu. The closed-vessel microwave digestion system Ethos 1 (Milestone, Bergamo, Italy) was equipped with sensors for temperature and pressure control and provided with PTFE (polytetrafluoroethylene) vessels capable of withstanding pressures of up to 110 bar. Stock standard solutions of Cr, Cd, Pb, Cu, Zn, V, Co, and Bi (1000 mg L^−1^ in 2% nitric acid) were purchased from Fluka (Milan, Italy) and were used in preparing calibration solutions.

To test whole blood and serum, about 0.25 g were added with 1 mL of internal Re standard at 0.5 mg L^−1^, and were then digested with 7 mL of HNO_3_ (69%, *v/v*) and 2 mL of H_2_O_2_ (30%, *v/v*). Instrumental parameters and settings were 15 min for 1500 W up to 180 °C, 15 min for 1500 W at 180 °C. Samples of 10 g of the mane and tail hair were cut into fragments of approximately 0.3 cm and washed four times with 1:200 (*v/v*) dilution of Triton X-100 solution to remove exogenous elements, rinsed twice with isopropyl alcohol, and allowed to drain. This was followed by rinses with Milli-Q water and two more rinses with acetone, and they were then allowed to drain. For hay, concentrate, and hair analyses, about 0.50 g of sample was added to 1 mL of internal Re standard and then digested with 7 mL of HNO_3_ (69%, *v/v*) (J.T. Baker, Milan, Italy) and 2 mL of H_2_O_2_ (30%, *v/v*) (J.T. Baker, Milan, Italy). Instrumental parameters and settings were 10 min for 1200 W up to 200 °C, 10 min for 1200 W at 200 °C. After being allowed to cool, each sample was made up to 25 mL volume using ultrapure water. Blank solutions were processed in the same way and were run with each batch of digested samples. The spiked samples used in the validation studies were digested under the same conditions as the samples. The samples of water were diluted and acidified with 2% nitric acid. The samples were filtered with 0.45 µm filter to remove larger particles. All determinations were carried out in triplicate. Samples were analyzed in batches, with blank samples and known standards and analyses carried out in triplicate according to Di Bella et al. [2]. The technique used, particularly with multiple simultaneous mineral analyses, removes almost all interference. However, depending on the type of study, potential interferences should be particularly considered for the analysis of cadmium and, possibly, other minerals when analyzed simultaneously and not individually.

#### 2.1.3. Statistical Analysis

The data obtained were analyzed statistically with GraphPad Prism version 10 (GraphPad Software Inc., La Jolla, CA, USA). The data were tested for normal distribution with the Kolmogorov–Smirnov test (*p* < 0.05) and they were represented as mean ± standard deviation (SD). The differences among trace elements (V, Cr, Co, Cu, Zn, Cd, Pb, and Bi) in different biological components (whole blood, serum; mane and tail) were statistically evaluated by the application one-way analysis of variance (ANOVA). The relationships among all elements in all biological substrate (whole blood, serum; mane and tail) were examined by linear regression analysis, and the correlation was expressed by Pearson’s correlation coefficient. Statistical significance was set at *p* ≤ 0.05.

## 3. Results

The concentrations (mean ± SD) of V, Cr, Co, Cu, Zn, Cd, Pb, and Bi contained in feed (hay and concentrate) and in water administered to horses are reported in Table 1. 

Shown in Table 2 are the statistical results for the studied mineral elements (mean ± SD) in biological components (whole blood, serum, mane, and tail) of horse. All elements showed statistically significant differences compared to all the other different biological components (*p* < 0.01; Table 2). 

**Figure 1:**
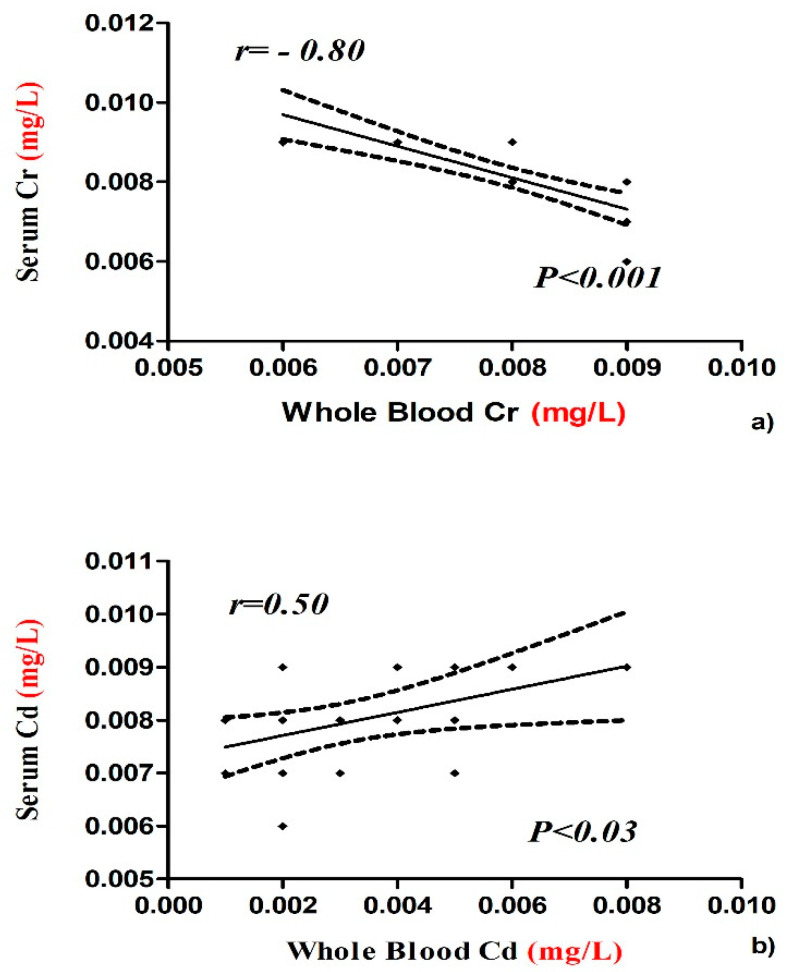
Negative correlation between whole blood and serum concentration of Cr (**a**) and positive correlation between whole blood and serum concentration of Cd (**b**) with Pearson’s correlation coefficients and significant values in horse.

**Figure 2:**
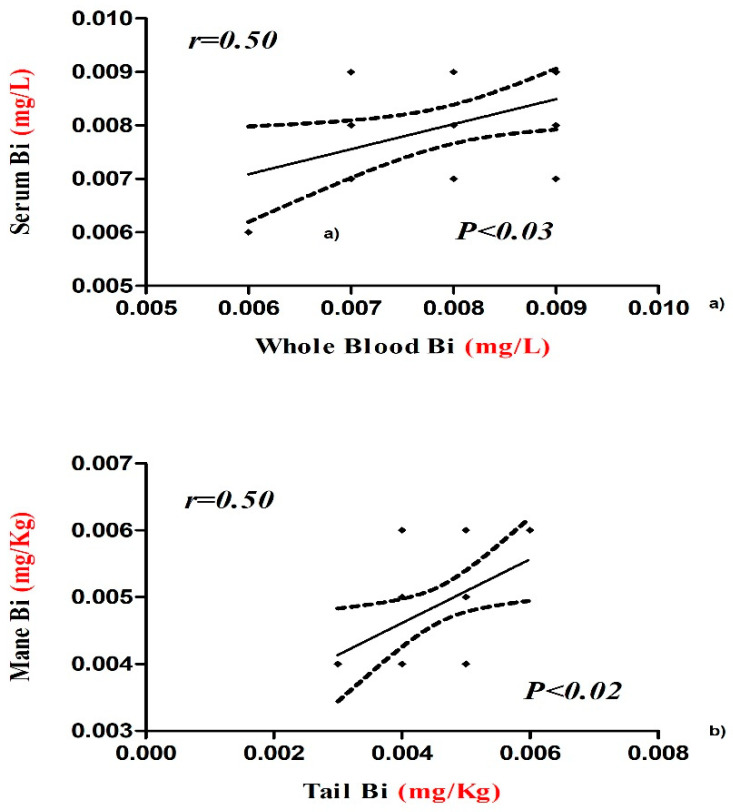
Positive correlation between whole blood and serum concentration (**a**) and mane and tail hair concentration (**b**) of Bi with Pearson’s correlation coefficients and significant values in horse.

**Figure 3:**
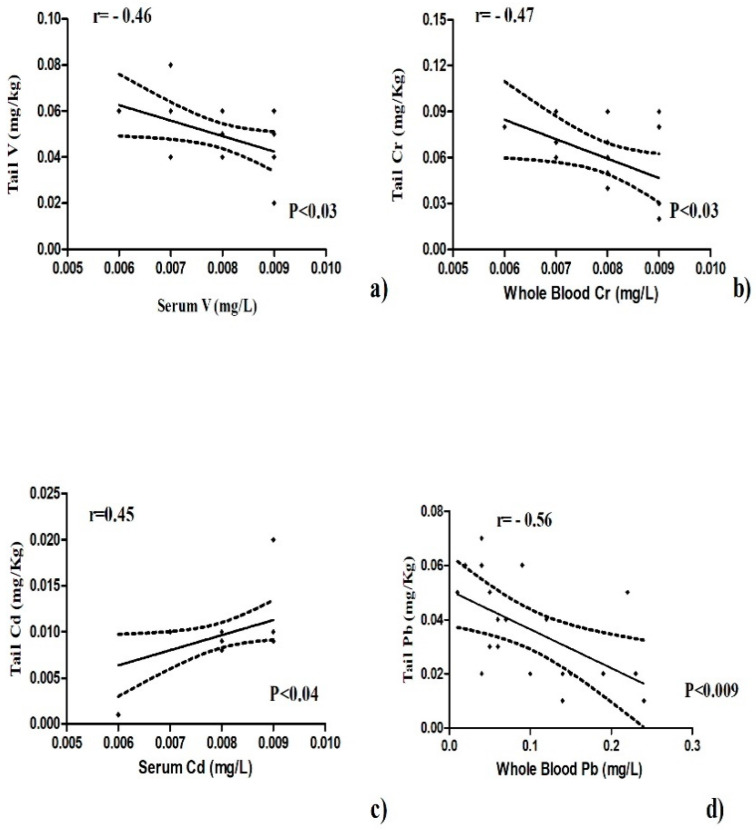
Negative correlation of V, Cr, and Pb in tail with serum (**a**) and whole blood samples (**b**,**d**). Positive correlation of Cd in tail with serum (**c**) shown as Pearson’s correlation coefficients and significant values.

The concentrations of Cr and Cd in whole blood and serum samples were negatively and positively correlated, respectively (Figure 1). Bismuth value showed a positive correlation between serum and whole blood and between tail and mane (Figure 2). The concentrations of V, Cr, and Pb in tail were negatively correlated with serum and whole blood samples (Figure 3a,b,d), while the concentrations of Cd in tail and serum samples were positively correlated (Figure 3c).

## 4. Discussion

The concentrations of the mineral elements present in fed hay and concentrate were below the limits established by the EC (European Commission, 2006) and potential toxic intake levels reported for horses as shown in Table 1. Our results show statistical differences in the concentration of chemical elements among the tested biological substrates (Table 2). Some authors showed the importance of mineral elements in health and disease conditions of humans, animals, and plants in order to prevent diseases related to bioaccumulation [12]. The organism uses various biochemical mechanisms to sequester this chemical element in order to minimize their potential toxicological impact, so hair should represent an important biological substrate for bioaccumulation. It was demonstrated that the level of bioaccumulation of some heavy metals (Pb, Cd, Hg, As) in horse depended on age [13]. Hair analysis can be useful to screen for long-term exposure to various chemical element in the organism and a major advantage is that it is non-invasive, does not degrade or require refrigeration, and little technical skill is required to obtain a sample [14]. The intoxication of horses by heavy metals is of comparatively uncommon occurrence, and the diseases are mostly chronic in type [15]. Casteel (2001) [16] review analyses in horse regarding the possible pathological effects of anthropogenic load of heavy metals and, in particular, about diseases related to musculoskeletal system and kidneys. The concentrations of V, Cu, Zn, and Cd showed significantly lower values in serum samples compared to whole blood and higher in the tail compared to the mane. Vanadium compounds are widely distributed near polluted areas because of its wide application in the chemical industry which increases the possibility of vanadium environmental exposure [17]. Concentration of this element in blood and serum was the most suitable indicator of bioaccumulation and was better tolerated by small species than by larger animals, such as horses [18]. Reference values for vanadium in whole blood and serum are not found in the literature. Our results reported a higher V serum concentration with respect to whole blood and these data were lower than those reported by Nazifi et al. [19], which does not indicate differences in whole blood levels due to gender and age of horses. Once in the bloodstream, V is distributed and stored in different tissues and among the various tissues, with the bioaccumulation in hair being of interest as it represents a biological substrate that is easy to collect [20]. In humans, some studies have shown V concentrations in the scalp hair of healthy adults due to potential bioaccumulation of this element, and in horses, no study has been conducted to evaluation bioaccumulation in hair [21]. Our results show higher V levels in whole blood and mane than serum and tail. The same V concentration was observed in whole blood and tail; this allows hypothesizing of an alternative use of the tail for the determination of bioaccumulation of this element in horses. Copper and zinc are important microelements for animal and human organisms because of their great positive role in physiological and regulatory processes, and they are required for the metabolic activities of numerous metalloenzymes. However, due to their redox activity, these elements can be toxic [22,23,24]. Our results show higher Cu and Zn concentrations in whole blood than the other tested biological substrates (Table 2), and Cu content in serum was affected by areas and seasons. In each biological substrate, the mean Cu concentrations were within the normal range expected for horses, with a value between 1.04 and 1.77 µg/mL as reported by Wichert et al. [25], 0.5–1.5 μg/mL. Copper levels in main and tail samples were lower than reported by de Souza et al. [10], in which a mane and tail pool was analyzed. Therefore, bioaccumulation was not observed for this element. Zinc content in whole blood samples was statistically higher (4.74 ppm) than in serum, tail, and mane. It is interesting to underline that in all tested biological substrates, Zn levels were higher than the reference values (0.77–1.19 μg/mL) defined by Thompson (1992) for horses or those adopted (0.6–1.2 μg/mL) with 106 horses from Bavaria (Thompson [10]). The bioaccumulation of this element in the organisms could be due to various factors and probably indicate smoke from galvanizing factures. Anyway, the studied athletic horses did not show any clinical signs of zinc intoxication. Therefore, the reported data can be considered a new reference range in horses kept in Messina. Cadmium showed statistically significantly higher values in whole blood than the other tested biological substrates. Its concentration in whole blood was positively correlated with that of serum, as shown in Figure 1. The mean Cd content in the blood samples of the present study (0.0031 μg mL) was below the range (0.005 μg/mL; 0.0075 to 0.0158 μg/mL) observed in horses in some polluted areas in Romania and then the value shown by Souza et al. (2014) [10]. In addition, cadmium concentrations in mane and tail were lower than reported by Souza et al. (2014) [10] in horse hair. Clinical signs of Cd intoxication are not common, but were observed by Liu (2003) [24] in animals raised near an industrial area. The horses are valuable as models for research on humans in relation to the effects of Cd on metabolism and in the potential accumulation of Cd content in blood representing a useful bioindicator of environmental pollution. There were statistically significant differences among the cobalt concentrations in the various tested biological substrates. Higher values were observed in tail (0.010 ppm). The observed Co levels were lower than indicated by Knych et al. (2015) [26] of around 1 part per billion (ppb). Studies showed that cobalt accumulates in body tissues over time, leveling off between 9 and 33 days in horses [27]. Because cobalt has a long half-life of about 6 ½ days, testing can readily reveal abnormally high concentrations in blood [26]. Regarding Cr concentrations, these did not vary significantly in whole blood, serum, and mane samples, while in the tail, the values were significantly higher than for all the other substrates. Cr levels were found to be inversely correlated between blood and serum (Figure 1). One explanation might be that different ionic forms of Cr are present in the measurement performed and that those are differently distributed between whole blood and serum [7]. In blood and serum samples, chromium concentration was low with respect to human serum chromium levels that are normally less than or equal to 1.4 µg/L [24]. No reference values are reported for horses. The concentrations of Cr and Pb in tail were negatively correlated with whole blood sample (Figure 3b,d), while the concentrations of V in tail were negatively correlated with serum (Figure 3a). For these elements, the tail hair sample does not seem to represent a valid alternative to the whole blood and serum sample for the evaluation of bioaccumulation in horse. By contrast, the concentrations of Cd in tail and serum samples were positively correlated (Figure 3c), and for these biological substrates, the potential bioaccumulation in horse could be evaluated in the future. The mean concentrations of Pb in whole blood and serum of horse show a more significant increase compared to mane and tail hair (Table 2). In animals, the Pb blood concentration that induces toxicity is much lower than that reported for humans, and our data in all tested biological substrates are within the range accepted for horses (0.25 µg/L). Dey and Dwived (2004) [28] evaluated 288 horses from an industrial area of India divided into three subareas (industrial, highway adjacent, and rural zone), and the mean Pb levels in serum were 0.47, 0.55, and 0.38 µg/L, respectively. Some authors have shown an association between hair Pb concentration and contamination in horse, and so they suggested the potential use of hair as a bioindicator for environmental pollution [25]. The value of Pb concentrations in mane and tail hair of our study do not support this hypothesis. With regard to Bi levels, no significant differences were observed between blood and serum and between mane and tail, while concentrations were significantly lower in the hair than in the blood and serum. The concentrations of Bi found in our samples were <0.008 mg/kg and, therefore, did not represent a toxicological risk for the studied horses. This element showed a positive correlation between whole blood and serum and between mane and tail (Figure 2), and the data obtained suggests the use of all tested biological substrates for the assessment of Bi bioaccumulation in horses. The toxicity of bismuth in animal models has been shown to destroy testicular macrophages, which leads to lower testosterone levels and reproductive dysfunction [29]. Ingested Bi is not absorbed by the human body and passes through the stool unaltered, and the small percentage that is absorbed into the body through the gastrointestinal tract is distributed in all body tissues with slightly higher concentrations in the liver and kidneys. The absorbed Bi is not metabolized and is eliminated from the body through kidney or liver processes [27]. Normal serum bismuth levels are between 10 and 50 μg/L, and toxicity rarely occurs if the serum concentration is below 50 ug/L (Micromedex 2011) [27].

## 5. Conclusions

This experience in the trace element analysis of biological fluids and other substrates suggests that high-resolution ICP–MS, which makes it possible to almost completely remove interference, and ETAAS with Zeeman correction of background absorption are the most effective techniques.

In this work, athletic horses were used that train in an area where the level of pollution from mineral elements can be high, and this could certainly be interesting to understand the possible decline in the athletic performance of horses due to the negative effects on hematological parameters. Chronic exposure, even at low concentrations of these substances, can cause cardiovascular toxicity, reproductive and developmental toxicity, neurotoxicity, nephrotoxicity, immunotoxicity, and carcinogenicity. 

The determination of essential trace elements in biological samples is an essential tool that suggests information that can be related to animal health and welfare. In particular, blood and serum are two matrices widely used in monitoring studies, but in the future, new biological substrates such as hair could represent a valid alternative used with a reliable method for biomonitoring in domestic animals. However, further investigations are needed on other species and in different biological substrates in order to have complete “body mapping” of the bioaccumulation of different essential trace elements.

## Figures and Tables

**Table 1:** Heavy metal parameters (mean ± SD) of horse-fed hay and concentrate together with the water values (mg/Kg of dry weight).

Heavy Metal Concentration in Feed and Water			
	Hay	Concentrate	Water
Vanadium	0.053 ± 0.005	0.0022 ± 0.0003	0.008 ± 0.001
Chromium	0.043 ± 0.006	0.010 ± 0.001	0.008 ± 0.001
Cobalt	0.04 ± 0.04	0.01 ± 0.00	0.008 ± 0.001
Copper	0.28 ± 0.02	0.19 ± 0.01	0.008 ± 0.001
Zinc	1.02 ± 0.10	0.73 ± 0.01	0.003 ± 0.0002
Cadmium	0.004 ± 0.005	0.001 ± 0.0005	0.007 ± 0.001
Lead	0.0233 ± 0.01	0.0041 ± 0.0003	0.008 ± 0.001
Bismuth	0.004 ± 0.001	0.004 ± 0.0003	0.009 ± 0.001

**Table 2:** Statistical results (mean ± SD) for the heavy metal parameters in biological component of horse. Values with different superscript (^a,b,c,d^) letter in the same row for each parameter are significantly different (*p* < 0.001).

Heavy Metal Concentration in Biological Substrate (mg/kg of Dry Weight for Mane and Tail)				
	Whole Blood	Serum	Mane Hair	Tail Hair
Vanadium	0.05 ± 0.01 ^a^	0.008 ± 0.001 ^b^	0.012 ± 0.01 ^b^	0.050 ± 0.013 ^a^
Chromium	0.008 ± 0.001 ^a^	0.008 ± 0.001 ^a^	0.017 ± 0.01 ^a^	0.060 ± 0.02 ^b^
Cobalt	0.005 ± 0.0029 ^a^	0.008 ± 0.001 ^b^	0.001 ± 0.0005 ^c^	0.010 ± 0.004 ^d^
Copper	1.47 ± 0.51 ^a^	0.68 ± 0.22 ^b^	0.11 ± 0.03 ^c^	0.15 ± 0.08 ^cd^
Zinc	4.74 ± 0.79 ^a^	2.16 ± 0.64 ^b^	1.86 ± 0.34 ^b^	2.07 ± 0.18 ^b^
Cadmium	0.0031 ± 0.0020 ^a^	0.008 ± 0.001 ^b^	0.002 ± 0.001 ^a^	0.010 ± 0.003 ^b^
Lead	0.10 ± 0.07 ^a^	0.11 ± 0.07 ^a^	0.02 ± 0.01 ^b^	0.04 ± 0.02 ^b^
Bismuth	0.008 ± 0.001 ^a^	0.008 ± 0.001 ^a^	0.005 ± 0.001 ^b^	0.005 ± 0.001 ^b^
